# Identification of Altered Developmental Pathways in Human Juvenile HD iPSC With 71Q and 109Q Using Transcriptome Profiling

**DOI:** 10.3389/fncel.2018.00528

**Published:** 2019-01-18

**Authors:** Karolina Świtońska, Wojciech J. Szlachcic, Luiza Handschuh, Paweł Wojciechowski, Łukasz Marczak, Michał Stelmaszczuk, Marek Figlerowicz, Maciej Figiel

**Affiliations:** ^1^Institute of Bioorganic Chemistry, Polish Academy of Sciences, Poznań, Poland; ^2^Institute of Computing Science, Poznan University of Technology, Poznań, Poland

**Keywords:** polyglutamine (polyQ), neurodevelopmental disease, iPSC, stem cells, neurodegenerative, Huntington disease, HD, RNA sequencing

## Abstract

In Huntington disease (HD) subtle symptoms in patients may occur years or even decades prior to diagnosis. HD changes at a molecular level may begin as early as in cells that are non-lineage committed such as stem cells or HD patients induced pluripotent stem cells (iPSCs) offering opportunity to enhance the understanding of the HD pathogenesis. In addition, juvenile HD non-linage committed cells were previously not directly investigated in detail by RNA-seq. In the present manuscript, we define the early HD and juvenile HD transcriptional alterations using 6 human HD iPS cell lines from two patients, one with 71 CAGs and one with 109 CAG repeats. We identified 107 (6 HD lines), 198 (3 HD71Q lines) and 217 (3 HD109Q lines) significantly dysregulated mRNAs in each comparison group. The analyses showed that many of dysregulated transcripts in HD109Q iPSC lines are involved in DNA damage response and apoptosis, such as CCND1, CDKN1A, TP53, BAX, TNFRSF10B, TNFRSF10C, TNFRSF10D, DDB2, PLCB1, PRKCQ, HSH2D, ZMAT3, PLK2, and RPS27L. Most of them were identified as downregulated and their proteins are direct interactors with TP53. HTT probably alters the level of several TP53 interactors influencing apoptosis. This may lead to accumulation of an excessive number of progenitor cells and potential disruption of cell differentiation and production of mature neurons. In addition, HTT effects on cell polarization also demonstrated in the analysis may result in a generation of incorrect progenitors. Bioinformatics analysis of transcripts dysregulated in HD71Q iPSC lines showed that several of them act as transcription regulators during the early multicellular stages of development, such as ZFP57, PIWIL2, HIST1H3C, and HIST1H2BB. Significant upregulation of most of these transcripts may lead to a global increase in expression level of genes involved in pathways critical for embryogenesis and early neural development. In addition, MS analysis revealed altered levels of TP53 and ZFP30 proteins reflecting the functional significance of dysregulated mRNA levels of these proteins which were associated with apoptosis and DNA binding. Our finding very well corresponds to the fact that mutation in the HTT gene may cause precocious neurogenesis and identifies pathways likely disrupted during development.

## Introduction

Huntington disease (HD) is a fatal dominantly inherited neurodegenerative disorder, caused by expansion of cytosine-adenine-guanine (CAG) repeats in exon 1 of the huntingtin (*HTT*) gene, resulting in elongated polyglutamine tract in HTT protein (MacDonald et al., 1993). HD symptoms are characterized by a general lack of coordination, progressive motor dysfunction accompanied by cognitive decline, psychiatric disturbances all leading to dementia (Victorson et al., 2014). The pathology of HD is most extensively localized in the central nervous system and includes striatal neuronal cell death followed by atrophy of the cerebral cortex, subcortical white matter, thalamus, hypothalamus, and other brain regions (Jimenez-Sanchez et al., 2017). Despite the fact that HD has a much-delayed clinical onset, subtle symptoms in patients may occur years or even decades prior to diagnosis. HD symptoms often aggravate with increasing number of CAG repeats and number of CAG repeats above 60 may result in disease onset occurring early in life, before age of 20 or even in childhood manifesting as juvenile HD (JHD) (Quarrell et al., 2013). Lack of HTT protein in an embryo is lethal probably because of HTT essential function in gastrulation (Saudou and Humbert, 2016) and for development of the nervous system, among others for development of brain corticostriatal pathways (Godin et al., 2010). Moreover, *HTT* gene is involved in the regulation of various biological processes and cellular activities that are impaired in HD cells, for example, apoptosis, transcription, signal transduction, vesicle-mediated transport, cytoskeleton assembly, centrosome formation and mitochondrial activity (Saudou and Humbert, 2016). In addition, a growing number of evidence points to HD as a neurodevelopmental disorder (Wiatr et al., 2018). In such context, the pathogenic function of mutant HTT in embryonic cells is not fully understood. The molecular HD changes begin at early cellular stages, even in cells that are not yet lineage committed such as stem cells. The molecular changes in the HD iPSC lines included MAPK signaling, increase in *SOD1* expression and decreased expression of *TP53* (Szlachcic et al., 2015) and changes of neurodevelopmental pathways (Ring et al., 2015). To date, high throughput transcriptional profiling focused on neuronal stem cells derived from HD patient iPSCs and demonstrated HD dysregulated genes and pathways, connected with GABA signaling, axonal guidance and calcium influx (HD iPSC Consortium, 2012, 2017). Until now, the single research group reported RNA-seq data on undifferentiated human HD iPSCs with 71 CAG repeats (Ring et al., 2015). However, no reports compared cells from juvenile patients with different number of CAG repeats and age of disease onset. A focus on pluripotent juvenile HD cells with a distinct number of CAG will be valuable for understanding the earliest events in HD pathogenesis and their impact on later developmental events and HD clinical picture. For example, it is unknown if pathways dysregulated already in stem cells may contribute to cell fate specification failures in HD.

We aimed here to reveal transcriptional changes in juvenile HD iPSC lines in order to identify dysregulated transcripts that may be involved in pathways critical for the early, neurodevelopmental HD pathogenesis. Therefore, we investigated the transcriptional profiles of several lines of HD juvenile iPSC with 71 and 109 CAG repeats using RNA-seq. We identified commonly dysregulated genes for both HD71Q and HD109Q iPSC lines and also unique genes dysregulated in sets HD lines with different CAG repeats. The mRNA profiling was followed by qRT-PCR validation of several mRNAs and bioinformatics analyses and also the mass spectrometry assay of proteins. As a result, we pointed out the involvement of several dysregulated transcripts and proteins in several biological processes crucial for proper neurodevelopment. In view of these results, it can be assumed that the molecular processes underlying juvenile HD begin as early as in stem cells in initial stages of embryo development.

## Materials and Methods

All experiments were conducted in accordance with the relevant guidelines and established standards.

### Human HD iPS Cells Culture

Human episomal HD and control iPSC lines were obtained from the NINDS Human Genetics Resource Center DNA and Cell Line Repository^1^. For the analysis, we used three clonal HD lines with 71 CAG repeats (ND42228, ND42229, ND42230; derived from a 20-year-old patient), three juvenile HD clonal lines with 109 CAG repeats (ND42222, ND42223, ND42224; derived from a 9-year-old patient) control lines (two clonal lines with 17/18 (ND41654, ND41658) and one line with 21 (ND42245) CAG repeats. Human iPSCs were cultured in chemically defined conditions in Essential 8 medium (Life Technologies) and grown on recombinant human vitronectin-coated surfaces (VTN-N, Life Technologies). Cells were passaged using gentle dissociation with 0.5 mM EDTA in PBS.

### RNA Isolation and Assessment

After medium removal, iPS cells were washed once with PBS and subsequently covered with 1 mL of RNAzol RT RNA Isolation Reagent (GeneCopoeia, Inc.), scraped and frozen in -80°C. Upon thaw, total RNA isolation was performed according to the manufacturer’s protocol with 75% ethanol, isopropanol, and RNase-free water. Isolated total RNA was then treated with TURBO DNase (ambion) and purified with QIAquick^®^ Nucleotide Removal Kit (QIAGEN). Each reaction was performed in PCR probes and contained 10 μg of RNA sample, 10x TURBO Buffer, RNaseOUT (Invitrogen), DNase TURBO and RNase-free water. The reaction mix was incubated in 37°C for 30 min and transferred to 1.5 ml Eppendorf tubes. Purification was performed according to the manufacturer’s protocol. RNA content was measured on NanoDrop. RNA quality and integrity were validated using capillary electrophoresis (2100 Bioanalyzer, Agilent). Average RNA Integrity Number (RIN) for all samples was 9.78, where 10 is the highest score.

### Library Preparation and RNA Sequencing

For RNA-seq, total RNA was extracted from 9 samples, including 3 from control iPSC lines, 3 from clonal iPSC lines with 71 CAG repeats and 3 from clonal iPSC lines with 109 CAG repeats. RNA was analyzed with Bioanalyzer 2100 and RNA 6000 Nano kit (Agilent Technologies, Santa Clara, CA, United States). Libraries were prepared from 5 μg of total RNA with RIN > 9.3, using KAPA Stranded mRNA-seq Kit (Kapa Biosystems, Wilmington, MA, United States) and NEBNext Multiplex Oligos for Illumina (New England Biolabs, Ipswich, MA, United States). 9 pM-indexed libraries were sequenced with the use of Illumina HiSeq2000, rapid run with paired-end 75 bp reads. On average, 65 mln reads were collected per library (Supplementary Figure S1).

### RNA-seq Data Statistical Analysis

NGSQCToolkit (v 2.2.3) (Patel and Jain, 2012) was used to generate quality metrics for assessment of FASTQ input files. Statistics generated by the above-mentioned tool was used to generate two charts which show the average quality score on the base positions for iPSC lines separately in R. Fastq files were then aligned to the GRCh38.p10 reference genome using STAR (v 2.5.3a) (Dobin et al., 2013) with default parameters suggested by QoRTs (v 1.3.0) (Hartley and Mullikin, 2015) used for genes and exons hits count. After alignment and hits counting, the differential gene expression was calculated using the Bioconductor package DESeq2 (v 1.14.1) (Love et al., 2014) and it consisted mainly of estimation of size factor and dispersion. The genes with significant differential expression were detected by DESeq2 pipeline and manually checked in order to find genes where outliers (automatic outliers detection with replacing their expression by mean across replicates is recommended for a larger number of replicates) influenced the mean expression of replicates. Dysregulated X and Y chromosome genes were excluded from DESeq2 analysis. After calculation of the differential expression by DESeq2, the shrinkage of effect size was performed (function lfcShrink of DESeq2 package). Then, for the results, the base mean expression against the log2 fold change (MA plots) were plotted with significant genes marked in red (plotMA from DESeq2 package). The gene counts were transformed to the log2 scale and normalized with respect to the library size (rlog function). Then, the Principal Component Analysis (PCA) was performed, and a chart was generated automatically by plotPCA DESeq2 function. For each normalized gene count for each sample the total mean gene expression was subtracted and based on the result, the heat map chart was prepared for manually selected list of genes (pheatmap function from pheatmap_1.0.10 package). All the RNA-seq data were uploaded to the GEO repository (accession number GSE124664).

### RT-PCR and Quantitative Real Time PCR

Reverse transcription was performed using Maxima H Minus Reverse Transcriptase (Thermo Fisher) (200U per reaction) on 2 μg of RNA in 20 μl of total reaction according to the manufacturer’s protocol. For priming a mixture of random hexamers (25 pmol) and oligo(dT) 18 (25 pmol) was used. Additionally, RiboLock RNase inhibitor was added to the reaction mix (20U). Before adding the enzyme and the inhibitor, templates were denatured in 65°C for 5 min; after mixing all reaction reagents reaction was incubated for 10 min at 25°C followed by 15 min at 50°C. Resulting cDNA was further 10 times diluted with nuclease-free water and stored in -20°C. RT- controls were included. RT-PCR products from all HD lines were checked for reference gene (*GAPDH*) and several pluripotency markers, including *SOX2, NANOG, OCT4* and *LIN28A* (Supplementary Figure S2).

qPCR was performed using HOT FIREPol^®^ EvaGreen^®^ qPCR Mix Plus (ROX) (SOLIS BIODYNE) on 1 μl of cDNA in 10 μl of total reaction volume. Reaction mix included 250/125 nM primers. Primers are listed in Supplementary Table S1. Thermocycling parameters were as follows: 12 min of initial denaturation at 95°C and 45 three-step cycles with 15 s denaturation at 95°C; 20 s annealing at 60–64°C and 20 s elongation at 72°C. The reaction was run on CFX96 instrument (Bio-Rad). Specificity of reaction for each primer pair was confirmed by agarose gel electrophoresis and EtBr staining. For each primer calibration curves were prepared within 10 1 to 10 -4 cDNA concentration; slope, y intercepts, PCR efficiency, r 2, linear dynamic range an CIs were calculated. Details for each primer set are included in Supplementary Table S1. Each qPCR plate included a calibrator, non-treated control (NTC) and samples were run in duplicates. For each cDNA template, a corresponding RT- sample was amplified; none of the analyzed samples had a positive signal of RT- reaction. Data was obtained and analyzed using CFX Manager 3.1 (Bio-Rad). Cq values were determined in software using the regression model and were exported to Excel for further analyses. *GAPDH*, *PGK1*, and *C1orf43* were used as reference genes.

Log2 NRQ was calculated as follows for each gene. First, a mean Cq was calculated for each sample as an arithmetical mean of technical replicates Cq values. Then the mean Cq values were corrected for a gene’s amplification efficiency by its multiplication by Log2 of *E*. *E* values was calculated as a (%Efficiency ^∗^ 0.01 + 1). Next, Relative Quantities (RQ) were calculated for each sample as the exponentiation of the efficiency corrected mean Cq of a sample subtracted from the arithmetic mean Cq of control samples mean Cqs, with the base of E, which is Eˆ[(arithmetic mean of control samples Cqs) – sample Cq]. Normalized Relative Quantities (NRQ) for each sample were calculated by dividing a gene of interest (GOI) RQ by Normalization Factor (NF). Normalization factor for each sample was calculated as a geometrical mean of Reference Genes (REF) RQs of that sample. Next, a base 2 logarithm was calculated from NRQ values and the resulting symmetrical Log2 NRQ value for each sample and GOI was visualized as a dot in a scatter plot together of Mean Log2 NRQ for each genotype. To calculate 95% CI in an RQ for genotype (geometrical mean of mean genotype Cqs) and genotype Standard deviation (from Mean genotype Cqs) were calculated. Based on these values NF and NRQ were calculated as before (genotype NF and genotype NRQ). Error propagation was calculated in subsequent steps: RQ SD = genotype SD ^∗^ genotype RQ ^∗^ ln(E), then SD NF = NF ^∗^ sqrt(sum(SD RQ/X^∗^ genotype RQ)ˆ2)REF1toX), where X is number of REF genes; SD NRQ = genotype NRQ ^∗^ sqrt((SD NF/NF)ˆ2 + (SD RQ/RQ)ˆ2), then SE NRQ = SD NRQ/sqrt(genotype N), where N is number of samples per genotype, then SE Log2 NRQ = SE NRQ/(genotype NRQ ^∗^ ln(2)). The final Combined-SE Log2 NRQ, which combines SE of a disease genotype with SE of a control genotype is sqrt ((SE log2 NRQ dis)ˆ2 + (SE log2 NRQ ctrl)ˆ2). Upper and lower 95% CI limits were as follows: CI = Log2 NRQ ± s^∗^Combined-SE Log2 NRQ, where s is a scaling parameter value from a t-Student table for N-1 degrees of freedom and 0.05 confidence.

### Bioinformatics Analysis

Two open source platforms were used for the bioinformatics analysis of significantly differentiated transcripts, including ConsensusPathDB (CPDB) (Herwig et al., 2016), and ClueGO (Cytoscape plug-in) (Bindea et al., 2009, 2013). A simple meta-analysis, considering data from several publications, was also performed. As considered to be significantly dysregulated, transcripts with padj < 0.05 were submitted to the analyses. As genes identifier type, HUGO Gene Nomenclature Comity symbols (HGNC) symbols were chosen.

#### Genetic, Biochemical, and Protein Interaction Analyses

A list of differentially expressed mRNAs was submitted to the web interface of CPDB for induced network modules analysis to create a network of different types of functional interactions between transcripts. CPDB collects data from 32 public resources, such as Kyoto Encyclopedia of Genes and Genomes (KEGG), Wikipathways, Reactome, Pathway Interaction Database (PID) and BioGRID, and integrate them to create networks containing different types of interactions.

We performed three separate analyses, one for each comparison group. We pasted a list of significantly dysregulated mRNAs in both HD lines and only in HD71Q or HD109Q lines. 63, 73, and 69% of accession numbers from the input lists were mapped to proteins in CPDB, respectively. Identifiers which were not mapped are listed in the Supplementary Table S2. In the next step, we chose protein, genetic, biochemical and gene regulatory interactions to be considered. For protein interactions confidence filter, we decided to choose high and medium confidence in the analysis considering both HD lines and high, medium and low confidence in the two following analyses. We set intermediate nodes z-score threshold to 30 in the analysis considering transcripts DE in both HD iPSC lines. Intermediate nodes were excluded from the analyses for genes dysregulated only in 71Q or 109Q HD lines. Nodes which were additionally added to the generated network and which were placed peripherally were deleted from the network in order to make the whole protein complex clearer. What is more, in order to visualize down- and upregulation in created networks we decided to upload log2FoldChange values for transcripts, obtained during the RNA-seq data statistical analysis. In order to do that, we chose the overlay values option.

#### Over-Representation and Enrichment Analyses

Over-representation and Gene Ontology (GO) enrichment analyses were conducted in CPDB to reveal overrepresented functional terms in the genomic background. Pathway-based sets and Gene Ontology-based sets were the two chosen categories of pre-defined gene sets among which over-represented terms were searched. Pre-defined sets contain proteins and/or genes that are together annotated with a specific pathway or GO term. A list of significantly dysregulated transcripts was submitted to the analysis (*p* < 0.05). For the pathway-based sets, search settings were set to 2 minimum overlap with input list and *p*-value cutoff = 0.01. Gene ontology level 4 and 5 were chosen to identify overrepresented biological processes, molecular functions, and cellular components. GO terms showing *p* < 0.01 were regarded as significantly enriched.

Enrichment analysis and data visualization were also performed with ClueGO app, which is another Cytoscape plug-in. It analyzes interrelations of terms and functional groups in biological networks. Gene identifier sets were directly uploaded in a simple text format. Few adjustments were made to reveal functional clusters for submitted transcripts. Analysis mode was set to “ClueGO: Function,” network specificity was set as default, between medium and detailed although much closer to the medium value. The visual style was set to “Groups.” We also selected “Use GO Term Fusion” function, which fuses GO parent-child terms based on similar associated genes, and “Show only Pathways with pV ≤ 0.05.” In the advanced term/pathway selection options, no changes were made. In the advanced statistical options enrichment/depletion (two-sided hypergeometric test) was chosen and the pV correction, which refers to the most significant pVs, was set to “Bonferroni step down.” The preferred layout was set to Organic Layout (yFiles).

#### Meta-Analysis of Differences in Gene Expression

Nine works listed in Table 1, all containing HT (microarray, RNA-seq) data on human HD cells, were selected for the meta-analysis. The analysis was performed similarly as previously (Wiatr et al., 2018), but with the inclusion of the additional data from the recent HD consortium publication (HD iPSC Consortium, 2017) and in order to compare dysregulated genes from these other publications with dysregulated genes obtained from our work. The names of the dysregulated genes or proteins were retrieved from 9 original publications and were sorted into 4 separate lists. We did not distinguish between the dysregulated genes/proteins identified in transcriptomic and proteomic experiments. Names of genes and names of genes corresponding to dysregulated proteins were subsequently listed as HGNC symbol. We established a list containing names of genes dysregulated in ESC, iPSC, NSC, and neurons. The genes overlapping between lists, and the genes reported in more than one of the nine studies included in the meta-analysis, were identified using MS Excel formulas.

**Table 1:** List of publications used for the meta-analysis of differences in gene expression.

**Publication reference**
Ring et al., 2015
HD iPSC Consortium, 2012
HD iPSC Consortium, 2017
An et al., 2012
McQuade et al., 2014
Chae et al., 2012
Nekrasov et al., 2016
Chiu et al., 2015
Feyeux et al., 2012


### Protein Extraction, Ultrafiltration, and Digestion for Proteomics Analysis

Each cell line was collected for lysis in buffer containing 1 M triethylammonium bicarbonate (TEAB) and 0.1% SDS. The material was subjected to a threefold cycle of freezing and thawing followed by bath sonication for 3-min repeated three times while cooling the tube on ice in between the sonication. The material was centrifuged in 8,000 × *g* for 10 min and the supernatant was collected. Lysates were then ultra-filtrated using Amicon Ultra-2 Centrifugal Filter 3 kDa Devices (MERCK) to remove any remnants of culture media. The device was pre-rinsed with milli-Q water and 600 μL of lysate was subjected to a fivefold cycle of centrifugation with a fresh amount of TEAB buffer in 7,500 × g for 5 min. Proteins in clear concentrate were estimated using 2-D Quant Kit (GE Healthcare Life Sciences). Ten μg of total protein per sample were diluted with 15 μl of 50 mM NH4HCO3, reduced with 5.6 mM DTT for 5 min at 95°C followed by alkylation with 5 mM iodoacetamide for 20 min in the dark at RT. Subsequently, the proteins were digested with 0.2 μg of sequencing-grade trypsin (Promega) overnight at 37°C followed by label free quantitative proteomics.

### Mass Spectrometry Analysis of Proteins

Analysis of protein extracts was done on Dionex UltiMate 3000 RSLC nanoLC System coupled with QExactive Orbitrap mass spectrometer (Thermo Fisher Scientific). Peptides derived from in-solution digestion of proteins were separated on a reverse phase Acclaim PepMap RSLC nanoViper C18 column (75 μm × 25 cm, 2 μm granulation) using acetonitrile gradient (from 4 to 60%, in 0.1% formic acid) at 30°C and a flow rate of 300 nL/min (for 185 min). Mass spectra were acquired in a semi-targeted method using two analysis modes. First was a classic data-dependent mode with top 10 data-dependent MS/MS scans, and second, was scheduled MS/MS mode with inclusion list containing peptide sequences chosen based on protein targets selected from previous transcriptome analysis. The target value for the full scan MS spectra was set to 1e6 with a maximum injection time of 100 ms and a resolution of 70,000 at m/z 400. The 10 most intense ions charged two or more were selected with an isolation window of 2 Da and fragmented by a higher energy collisional dissociation with NCE 28. The ion target value for MS/MS was set to 5e4 with a maximum injection time of 100 ms and a resolution of 17,500 at m/z 400.

### Analysis of Proteomic Data

Protein identification was performed using UniProt human database (March 2017, 137404 entries) with a precision tolerance 10 ppm for peptide masses and 0.08 Da for fragment ion masses. For protein identification and quantification, all raw data obtained were analyzed using MaxQuant 1.5.3.30 (Max Planck Institute of Biochemistry, Munich) (Cox and Mann, 2008). Obtained normalized data were imported to Perseus 1.6.1.3 software (Max Planck Institute of Biochemistry, Munich) (Tyanova et al., 2016). All numeric values corresponding to protein intensity were transformed to a logarithmic scale, and all samples were grouped using categorical annotation. Missing values were then replaced by imputation and PCA analysis was performed. For protein differentiation, ANOVA test was performed with the *p*-value calculation used for protein truncation. The proteins were annotated for chromosome localization and all proteins translated from genes located on X and Y chromosomes were removed from the list. Then, for the clustering analysis, the data were normalized for each compound using the Z-score algorithm.

## Results

### Early Transcriptional Changes in Human HD iPSC Lines Revealed by Next-Generation High-Throughput RNA Sequencing

**FIGURE 1:**
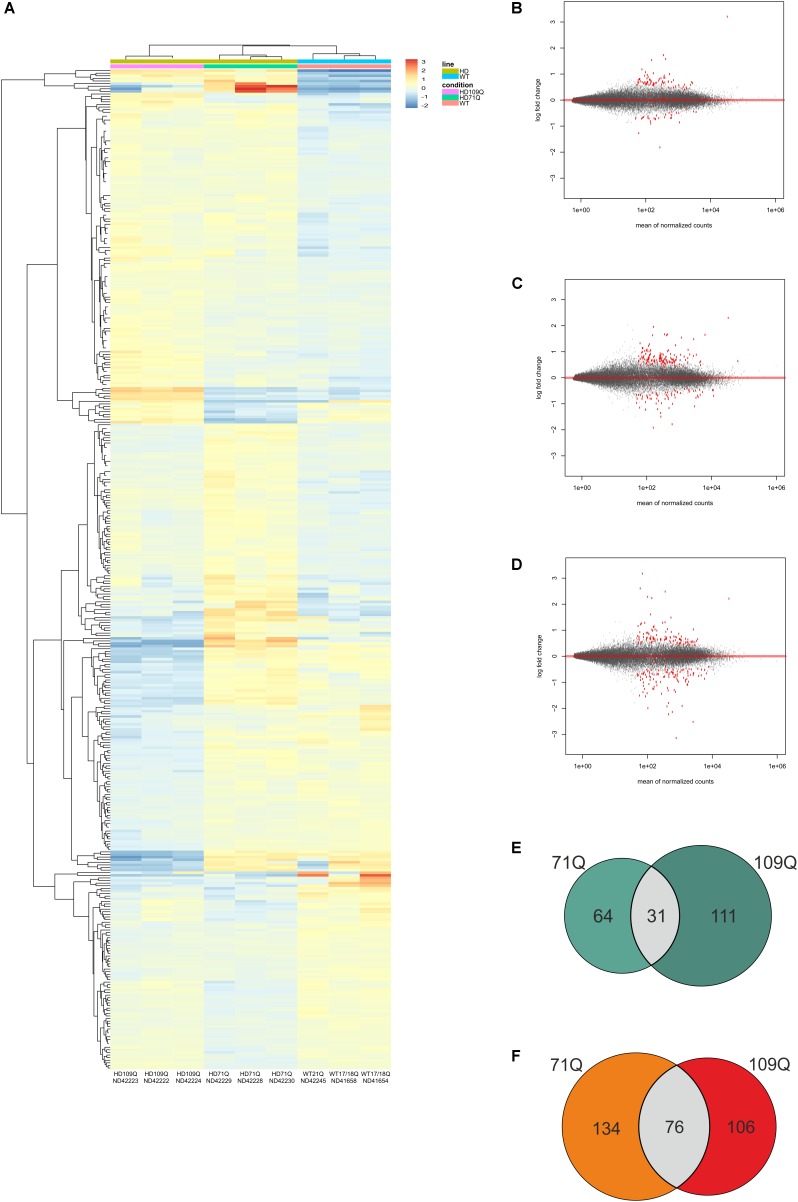
RNA-seq Analysis of HD and control iPSC lines show early transcriptional changes in affected cells. **(A)** Heat map representing gene expression patterns of DE genes when comparing HD71Q and HD109Q iPSCs to control iPSCs at an adjusted *p*-value of < 0.05 and baseMean cutoff > 50. Red represents elevated expression while blue represent decreased expression, compared with the row mean. Each column represents each isogenic line. Gene names are shown on Supplementary Figure S3. **(B–D)** MA plots – differential expression analysis of significantly downregulated or upregulated mRNAs in HD iPSCs vs. control lines, as determined by population level RNA-seq – for HD vs. WT, 71Q vs. WT and 109Q vs. WT, respectively. **(E)** Venn diagram display of downregulated genes. **(F)** Venn diagram display of upregulated genes.

Strand-specific RNA-seq of the whole transcriptome was performed using clonal lines from 2 HD patients with 71 or 109 CAG repeats in exon 1 of the HTT gene, and 2 healthy individuals, with 17/18 and 21 CAG repeats, to comprehensively identify mRNAs related to HD. A DESeq2 pipeline for transcripts of HD was developed to identify significantly dysregulated mRNAs. During the RNA-seq data analysis, we compared 6 HD iPSC lines derived from both patients (71Q and 109Q) with control lines and also compared separately each set of three HD lines from one patient with three control lines. As a result of such an approach, we generated statistical values for three comparison groups, HD vs. WT, HD71Q vs. WT, and HD109Q vs. WT (Supplementary Table S3). Heat map diagram, PCA graph and MA plots were generated for differential gene expression analysis (Figures 1A–D and Supplementary Figures S3, S4).

We set the padj cutoff value to create lists of significantly dysregulated mRNAs in each comparison group. Transcripts with padj < 0.05 were considered to be differentially expressed. In the first group, comparing HD iPSC lines (71Q and 109Q) with control lines, 107 significantly dysregulated mRNAs were identified (31 downregulated and 76 upregulated). In HD71Q iPSC lines 198 differentially expressed mRNAs were identified (64 downregulated and 134 upregulated). In the last group, in which HD109Q lines were compared with control lines, 217 significantly dysregulated mRNAs were identified (111 downregulated and 106 upregulated). The number of transcripts differentially expressed in both HD lines and in each line separately is shown on the Venn diagram (Figures 1E,F). We have focused our experimental validation and further bioinformatics analyses on these mRNAs (Supplementary Table S3). Lists of 30 the most dysregulated transcripts for each group were shown in Tables 2–4.

**Table 2:** Top 30 the most dysregulated mRNAs in HD iPSC lines (71Q and 109Q) when compared to control lines.

Symbol	Ensembl gene	Log2FoldChange	*p*-value	padj
*U1*	ENSG00000277918	6.253425519	7.44E-118	1.60E-113
*NANOGP8*	ENSG00000255192	4.839594108	1.77E-30	1.90E-26
*ALG10B*	ENSG00000175548	3.418278891	4.27E-26	3.06E-22
*CBSL*	ENSG00000274276	-3.09690354	8.47E-23	4.55E-19
*OTOGL*	ENSG00000165899	3.170966795	9.53E-20	4.10E-16
*TRIM69*	ENSG00000185880	1.140358901	1.69E-17	6.07E-14
*CNTNAP3B*	ENSG00000154529	-2.300972053	6.27E-12	1.93E-08
*TAS2R64P*	ENSG00000256274	1.893607772	1.09E-11	2.93E-08
*AC005276.1*	ENSG00000197462	4.247092094	1.45E-10	3.11E-07
*MEIOB*	ENSG00000162039	1.950419866	1.92E-09	2.96E-06
*LINC00649*	ENSG00000237945	1.07452898	2.69E-09	3.85E-06
*C3*	ENSG00000125730	1.347898641	6.03E-09	8.11E-06
*AC003973.3*	ENSG00000279377	11.45502355	2.81E-08	3.55E-05
*AC009005.2*	ENSG00000267751	1.298509518	6.23E-08	7.45E-05
*ZFP30*	ENSG00000120784	0.952711732	7.25E-08	7.80E-05
*RP11-78F17.1*	ENSG00000263551	1.583170794	6.94E-08	7.80E-05
*PARP12*	ENSG00000059378	0.870056271	8.53E-08	8.73E-05
*XDH*	ENSG00000158125	1.073538005	1.12E-07	0.000109785
*ZNF208*	ENSG00000160321	9.248212473	1.24E-07	0.000111532
*ZNF257*	ENSG00000197134	7.158769565	1.23E-07	0.000111532
*RP11-343H5.4*	ENSG00000224114	2.502023616	1.68E-07	0.000144269
*ACTA1*	ENSG00000143632	-2.644830326	4.07E-07	0.000316863
*RPL13P12*	ENSG00000215030	-2.978270182	4.10E-07	0.000316863
*RP11-114H24.2*	ENSG00000260776	1.437940024	4.12E-07	0.000316863
*RDM1*	ENSG00000278023	0.980241441	8.83E-07	0.000633438
*FAM86B3P*	ENSG00000173295	1.56869681	1.02E-06	0.000709353
*SLC24A3*	ENSG00000185052	-0.952696056	1.76E-06	0.001115434


**Table 3:** List of 30 the most dysregulated mRNAs in HD71Q iPSC lines compared to control lines.

Symbol	Ensembl gene	Log2FoldChange	*p*-value	padj
*U1*	ENSG00000277918	6.286072251	7.79E-87	1.68E-82
*NANOGP8*	ENSG00000255192	5.161894598	1.44E-32	1.55E-28
*ALG10B*	ENSG00000175548	2.883022339	3.12E-31	2.24E-27
*RNF20*	ENSG00000155827	-1.182765532	5.15E-25	2.77E-21
*PIWIL2*	ENSG00000197181	3.194234427	8.65E-23	3.73E-19
*OTOGL*	ENSG00000165899	3.434955786	1.73E-22	6.20E-19
*FAM65B*	ENSG00000111913	-2.775405407	8.52E-17	2.62E-13
*CBSL*	ENSG00000274276	-3.102564495	1.93E-16	5.20E-13
*APOBEC3B*	ENSG00000179750	-3.283380768	3.00E-16	7.19E-13
*MATN2*	ENSG00000132561	-1.152507133	5.20E-15	1.12E-11
*HIST1H3C*	ENSG00000278272	2.979803376	6.11E-15	1.20E-11
*ZNF257*	ENSG00000197134	8.043476357	4.82E-13	7.99E-10
*TRIM69*	ENSG00000185880	1.129527997	3.03E-12	4.35E-09
*HERC2P9*	ENSG00000206149	-1.392763638	4.07E-12	5.48E-09
*HIST1H2BB*	ENSG00000276410	2.83218348	6.10E-12	7.73E-09
*AC003973.3*	ENSG00000279377	12.38024867	9.33E-12	1.06E-08
*TAS2R64P*	ENSG00000256274	2.045242418	3.95E-11	4.25E-08
*GOLGA8B*	ENSG00000215252	-1.916866228	1.03E-10	1.05E-07
*CDKN1A*	ENSG00000124762	1.161235447	4.51E-10	4.22E-07
*CDH7*	ENSG00000081138	-1.86720424	4.50E-10	4.22E-07
*ZNF208*	ENSG00000160321	10.17366602	4.94E-10	4.43E-07
*LINC01535*	ENSG00000226686	3.470961414	8.70E-10	6.88E-07
*RP11-350D17.3*	ENSG00000271369	2.194112973	8.34E-10	6.88E-07
*CNTNAP3B*	ENSG00000154529	-2.50348849	8.95E-10	6.88E-07
*TTC13*	ENSG00000143643	-0.888229847	1.24E-09	9.21E-07
*ALS2*	ENSG00000003393	-0.742660378	2.71E-09	1.95E-06
*SULT1A1*	ENSG00000196502	-1.632429581	5.08E-09	3.53E-06
*AC016582.2*	ENSG00000225868	1.648898784	5.67E-09	3.82E-06
*MT-ND3*	ENSG00000198840	1.052399972	1.94E-08	1.23E-05
*THNSL2*	ENSG00000144115	-1.443657812	2.46E-08	1.51E-05


**Table 4:** List of 30 the most dysregulated mRNAs in HD109Q iPSC lines compared to control lines.

Symbol	Ensembl gene	Log2FoldChange	*p*-value	padj
*U1*	ENSG00000277918	6.219895444	4.67E-85	1.09E-80
*CDKN1A*	ENSG00000124762	-3.933483105	3.37E-73	3.93E-69
*PHLDA3*	ENSG00000174307	-2.551015174	3.38E-56	2.62E-52
*ALG10B*	ENSG00000175548	3.802701616	5.17E-54	3.01E-50
*TP53*	ENSG00000141510	-3.274551066	3.91E-48	1.82E-44
*EIF2S3L*	ENSG00000180574	3.037222044	6.21E-33	2.41E-29
*ASNSP1*	ENSG00000248498	5.965875222	4.48E-29	1.49E-25
*CDC20P1*	ENSG00000231007	4.12484708	6.13E-28	1.79E-24
*SNORD3B-1*	ENSG00000265185	-3.685369931	2.56E-27	6.64E-24
*INPP5D*	ENSG00000168918	-2.188602587	4.32E-27	1.01E-23
*MIR34A*	ENSG00000228526	-2.523159497	5.26E-26	1.11E-22
*NANOGP8*	ENSG00000255192	4.417935163	2.97E-24	5.77E-21
*MUC19*	ENSG00000205592	-3.336959246	3.06E-22	5.49E-19
*RP11-958N24.2*	ENSG00000227827	3.589013767	3.18E-21	5.30E-18
*BAX*	ENSG00000087088	-1.204461789	2.10E-20	3.26E-17
*LINC01021*	ENSG00000250337	-3.135350542	2.96E-18	4.31E-15
*CRYGEP*	ENSG00000229150	2.387074711	4.44E-18	6.10E-15
*CBSL*	ENSG00000274276	-3.091183178	1.72E-16	2.22E-13
*TEC*	ENSG00000135605	1.639741292	3.30E-16	4.05E-13
*OTOGL*	ENSG00000165899	2.83934664	8.89E-16	1.04E-12
*FAM86B3P*	ENSG00000173295	1.951641415	8.99E-14	9.98E-11
*RP11-366M4.11*	ENSG00000248632	-4.56130814	4.33E-13	4.59E-10
*RP11-632K20.7*	ENSG00000223509	1.925042321	8.28E-13	8.40E-10
*TRIM69*	ENSG00000185880	1.151508188	9.51E-13	9.24E-10
*SPATA18*	ENSG00000163071	-2.328900814	1.84E-12	1.72E-09
*RP11-115D19.1*	ENSG00000251095	-3.099666004	1.34E-11	1.11E-08
*AEN*	ENSG00000181026	-0.980726499	1.71E-11	1.38E-08
*HIST3H2BA*	ENSG00000181201	-1.810817061	3.56E-11	2.77E-08
*LIMCH1*	ENSG00000064042	2.500401748	3.98E-11	3.00E-08


**FIGURE 2:**
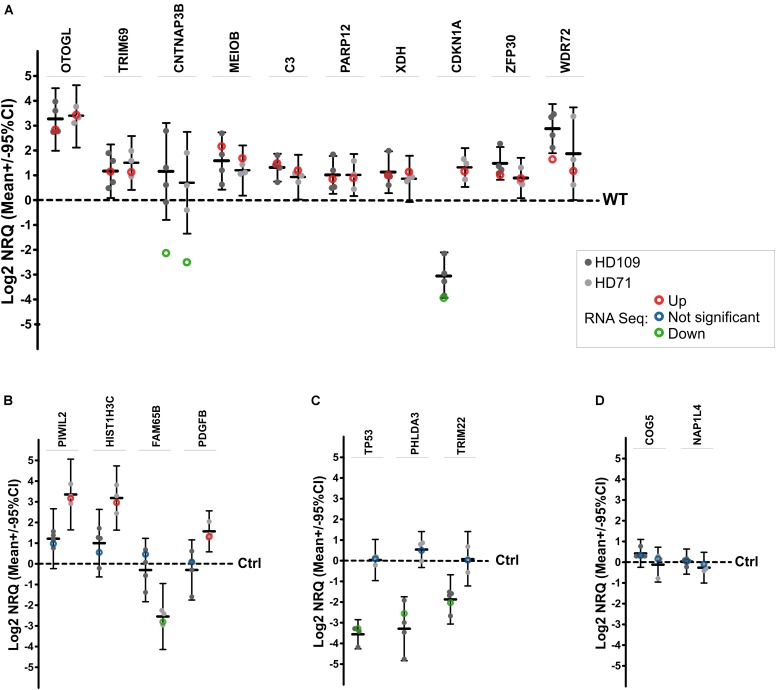
qPCR validation of RNA-seq results. Seventeen genes in total were selected for DE confirmation in the same RNA samples used for RNA-seq. Among selected genes, 10 were DE in both HD iPSC lines **(A)**, 4 were DE only in HD71Q iPSC lines **(B)**, and 3 genes were DE only in HD109Q iPSC lines **(C)**, compared to control lines. Results for each analyzed gene are presented as a scatter plot of Log2 NRQ values for each sample (*n* = 3 per genotype), together with Mean Log2 NRQ (thick horizontal line) for each genotype and 95% CIs (thin vertical lines). Dashed lines at 0 represent WT control lines. Genes are differentially expressed when 95% CI lines do not cross WT line. For easier reference, Log2 FC data from RNA-seq experiments (circles) were also included on plots. **(D)** Two additional reference genes, identified as not significantly dysregulated, were also selected for RNA-seq results validation.

A total of 17 differentially expressed mRNAs were selected to verify RNA-seq data by quantitative real-time PCR (qPCR) (Figure 2). We have selected 10 mRNAs which were differentially expressed in both HD iPSC lines (*OTOGL*, *TRIM69*, *CNTNAP3B*, *MEIOB*, *C3*, *PARP12*, *XDH*, *CDKN1A*, *ZFP30*, *WDR72*), 4 mRNAs which were differentially expressed in HD71Q iPSC lines (*PIWIL2*, *HIST1H3C*, *FAM65B*, *PDGFB*) and 3 mRNAs differentially expressed in HD109Q iPSC line (*TP53*, *PHLDA3*, *TRIM22*). Validation of transcripts dysregulated in both HD lines confirmed results obtained from the RNA-seq analysis for 7 out of 10 analyzed mRNAs. Validation of chosen transcripts dysregulated only in HD71Q lines was completely consistent with up- and downregulation in RNA-seq data. Last experimental analysis, considering mRNAs dysregulated only in HD109Q lines also showed consistency with RNA-seq (Figure 2).

### Altered Levels of Proteins Overlap With Several Dysregulated Transcripts in HD iPSC

**FIGURE 3:**
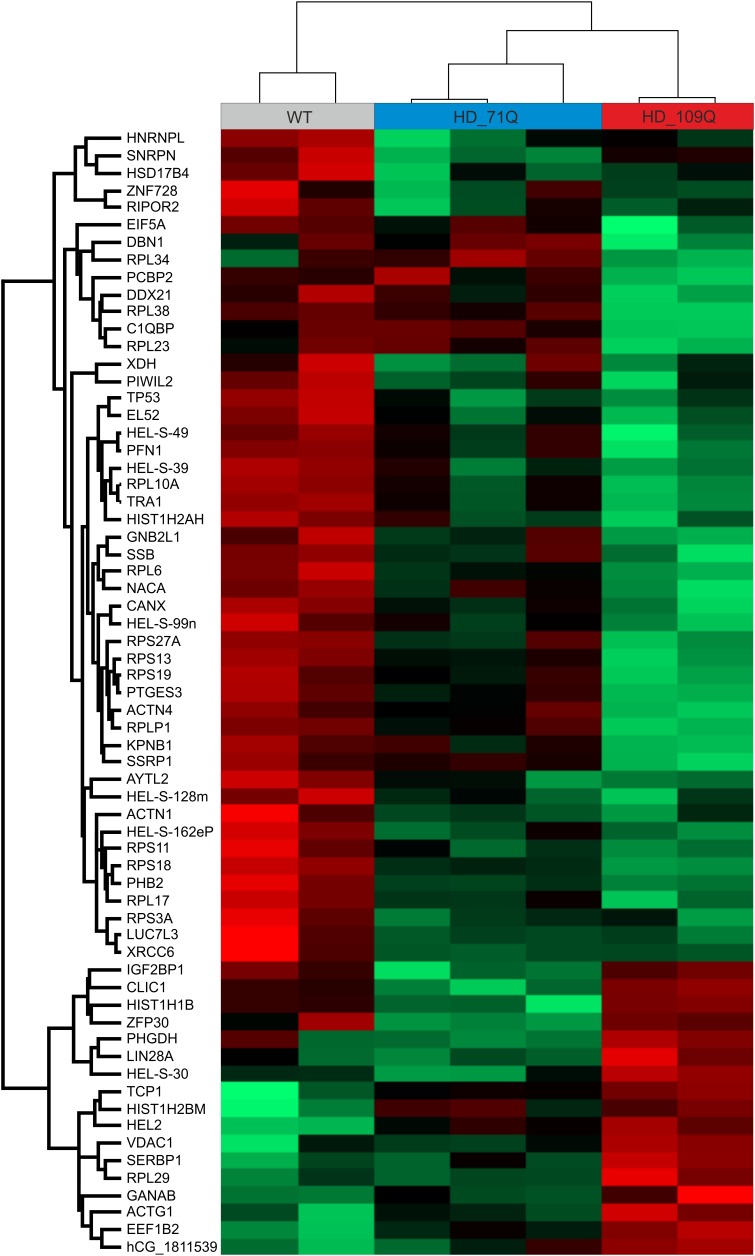
Mass spectrometry analysis of HD and control iPSC lines show early proteomic changes in affected cells. Heat map representing all differentiating proteins identified after comparing HD71Q and HD109Q iPSC lines to control iPSC lines. Red represents elevated level of protein while green represents decreased level of protein. Each column represents each isogenic line. Protein names are shown on the left side.

We performed a mass spectrometry analysis to validate dysregulated transcripts at the protein level (Figure 3 and Supplementary Table S4). As a result, we identified 65 differentiating proteins, however, the proteins from chromosomes X and Y were excluded. Among the proteins which demonstrated statistically significant change, there were TP53 and ZFP30 which were also found in the pool of dysregulated transcripts and which showed the same direction of the level change. These proteins are connected with intrinsic apoptotic signaling pathway in response to DNA damage (TP53) and DNA binding transcription factor activity (ZFP30). Moreover, in support of our transcriptional changes, we found that many of the non-statistically significant proteins demonstrated dysregulation pattern similar to significantly dysregulated transcripts.

### Interaction Analysis of HD-iPS Dysregulated Genes Identifies a Network Rich in Transcription Regulators in 71Q Lines, Whereas a Network of TP53-Dependent Genes in 109Q Lines

Using the web interface of CPDB we conducted the interaction- and pathway-centric analysis of list of differentially expressed mRNAs in each comparison group. Induced network modules analysis generates network in which genetic, biochemical and protein interactions between given genes are shown. It also includes genes that are not in the submitted list but connect two or more seed genes with each other and have many connections within the induced network module. Such bioinformatics approach helped us with visualizing biological associations between dysregulated transcripts and their connections with other genes, which may have an impact on HD pathogenesis.

**FIGURE 4:**
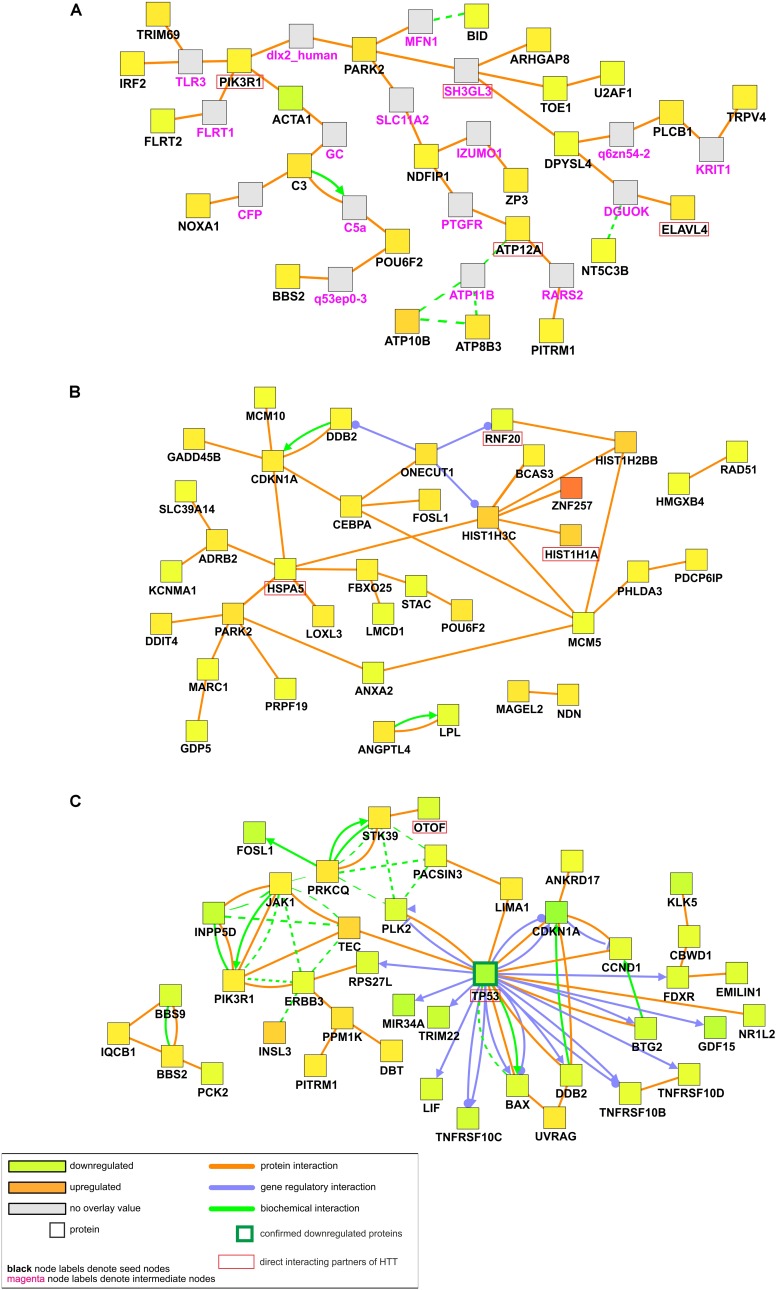
Induced network modules analysis of DE transcripts in HD iPSC lines. CPDB induced network modules aims to connect a list of seed genes via different types of interactions (protein interactions, biochemical interactions, or gene regulatory interactions). Connections are made directly, or via an intermediate node (shown in gray). As considered to be significantly dysregulated, transcripts with adjusted p value of < 0.05 were submitted to the analysis. As genes identifier types, HGNC symbols were chosen. **(A)** Network plot for significantly dysregulated transcripts in both HD iPSC lines vs. control. **(B)** Network plot for significantly dysregulated transcripts only in HD71Q iPSC lines. **(C)** Network plot for significantly dysregulated transcripts only in HD109Q iPSC lines. Proteins confirmed after proteomic analysis were pinpointed by a green border of the certain nodes. Names of direct interacting partners of the HTT protein were highlighted with red border.

Our first analysis considered mRNAs differentially expressed in both iPSC lines (Figure 4A). Proteins for 25 out of 107 dysregulated transcripts were assigned to the generated network. Main observation from the visualized protein complex is that most of seed genes are associated with each other through intermediate nodes which were not present in the input list. Although no obvious center node can be distinguished, PARK2 seems to be crucial for the whole network as it unites all of the other protein complexes. *PARK2* gene is associated with synaptic vesicle exocytosis and central nervous system development. Among other interesting proteins highlighted in the network, there is PIK3R1 whose gene is strongly upregulated in both HD iPSC lines and necessary for the insulin-stimulated increase in glucose uptake, but it is also connected with axon guidance and negative regulation of the apoptotic process. Different proteins in the generated network are also involved in signal transduction and nervous system development, like ARHGAP8, DPYSL4, and FLRT2. Other biological processes in which visualized proteins are involved are RNA splicing and cell motility.

Second analysis considered mRNAs differentially expressed only in HD71Q iPSC lines (Figure 4B). Proteins for 37 out of 198 seed genes were visualized during the analysis. The analysis shows the presence of histones (HIST1H3C, HIST1H2BB, HIST1H1A) and other transcription regulators (ZNF257, BCAS3) in the network. All of the downregulated genes for which proteins are present in the main network are also connected with transcription regulation, like *MCM5* and *RNF20*. Almost all of the upregulated nodes mentioned above are associated with positive regulation of transcription.

The third analysis, considering mRNAs differentially expressed in HD109Q iPSC lines (Figure 4C), shows the crucial role of downregulated transcripts and gene regulatory interactions. Proteins for 41 out of 217 genes from the input list were visualized in the network. The most important protein present in the generated network, with the highest number of edges, is TP53. Many of its interactors are, like TP53 itself, associated with apoptotic signaling, such as tumor necrosis factor receptor superfamily members, TNRFSR10B, TNFRSF10C, and TNFRSF10D but also BAX, CDKN1A, PLK2, HSPA1A, and others.

### Over-Representation and Enrichment Analyses Based on the Most Differentially Expressed Transcripts

Over-representation and enrichment analyses were performed with significantly dysregulated mRNAs for each comparison group. We used two bioinformatics tools to identify overrepresented gene ontology and pathway-based terms – CPDB and ClueGO.

**FIGURE 5:**
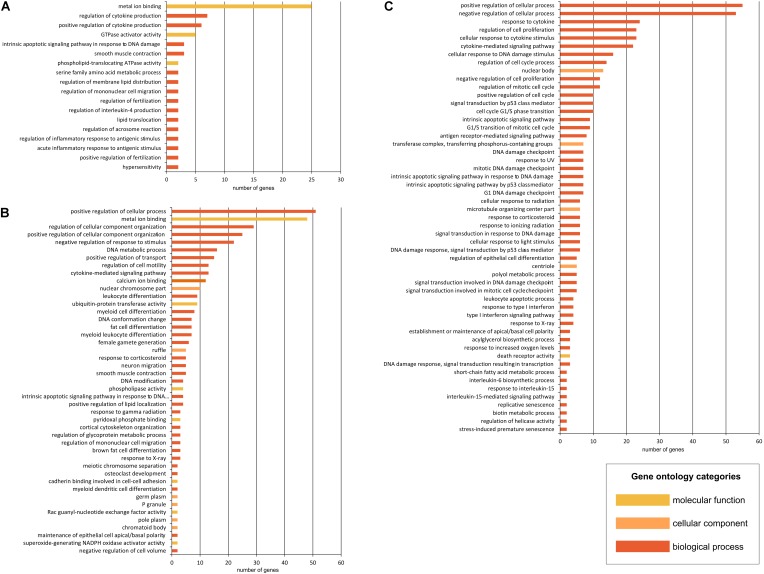
Gene Ontology (GO) term analysis in HD iPSC lines. In the CPDB over-representation analysis, the submitted list of dysregulated genes was mapped to physical entities. Gene Ontology-base sets, containing genes that are together annotated with a specific GO term, were generated for genes dysregulated in both HD iPSC lines **(A)** and for genes DE only in 71Q lines **(B)** or 109Q lines **(C)**. Functional sets were sorted by the decreasing number of genes in each set.

In each analysis, we have focused on identifying pathway and gene ontology-based gene sets considering molecular functions, biological processes and cellular components (Figure 5). First analysis included the list of differentially expressed mRNAs in both HD iPSC lines. The most overrepresented GO terms, with the biggest number of genes associated with the term were metal ion binding, regulation of cytokine production and GTPase activator activity (Figure 5A). As for the pathway-based analysis, phospholipase c signaling pathway was distinguished as the one with the lowest *p*-value (Supplementary Table S5). Phospholipase-C is known to be key signaling proteins in the cellular action of many hormones, neurotransmitters, growth factors, and other extracellular stimuli. The input overlap members in this pathway are *PLCB1* and *PIK3R1*. These two genes are also members of pathways associated with Joubert syndrome, a brain development disorder characterized by the agenesis or underdevelopment of the cerebellar vermis and also by the malformed brain stem. Other identified pathways include selective serotonin reuptake inhibitor pathway, reelin signaling pathway, Beta2 integrin cell surface interactions, downstream signaling of activated FGFR1 and apoptosis.

The most overrepresented GO terms obtained due to the analysis of significantly dysregulated transcripts in HD71Q iPSC line included positive regulation of cellular process, metal ion binding and regulation of cellular component organization (Figure 5B). Cellular components with the lowest *p*-value and the higher number of submitted transcripts at the same time referred to ruffle, germ plasm and chromatoid body. As for the pathway-based analysis, transcriptional regulation of white adipocyte differentiation is the one with the lowest *p*-value (Supplementary Table S5). The input overlap members in this pathway are *LPL*, *CEBPA*, and *ANGPTL4*. Other identified pathways are p73 transcription factor network, inflammatory mediator regulation of TRP channels, DNA damage response and lncRNA mediated mechanisms of therapeutic resistance.

Last analysis, which focused on differentially expressed mRNAs in HD109Q iPSC line, revealed the regulation of biological and cellular process and a great number of apoptosis-related terms (Figure 5C). As for the pathway-based analysis, direct p53 effectors and p53 signaling p53 signaling pathway are the most significant among identified pathways (Supplementary Table S5). Other include terms like validated transcriptional targets of TAp63 isoforms, DNA damage response, signaling pathways in glioblastoma, apoptosis modulation and signaling, Wnt signaling pathway and pluripotency and a few viral infection pathways.

Protein complex-based sets of mRNAs, whose protein products are members of the same annotated protein complex, are shown in Supplementary Table S6.

**FIGURE 6:**
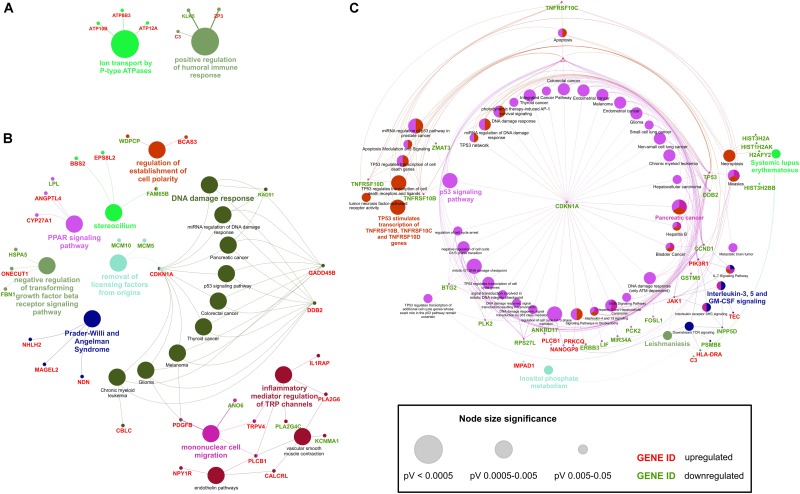
Overrepresentation and enrichment analysis of DE transcripts in HD iPSC lines. ClueGO (Cytoscape plug-in) analyzes interrelations of terms and functional groups in biological networks. Several functional clusters for submitted transcripts were identified. **(A)** Functional clusters generated with ClueGO analysis of transcripts differentially expressed in both HD iPSC lines vs. control. **(B)** Functional clusters for transcripts DE only in HD71Q iPSC lines. **(C)** Functional clusters for transcripts DE only in HD109Q iPSC lines. Genes shared between terms were also shown. Upregulation or downregulation of visualized genes was marked as red or green, respectively.

ClueGO overrepresentation and enrichment analysis of transcripts significantly dysregulated in both HD iPSC lines revealed positive regulation of humoral immune response and ion transport by P-type ATPases as GO terms with the lowest p value (Figure 6 and Supplementary Table S7) C3 gene, which is highlighted as associated with the first biological process, is in the top 10 dysregulated transcripts in both HD iPSC lines. Next to *C3*, there are few other genes that are present in many generated clusters, such as *ZP3*, *KLK5*, *ATP10B*, *ATP8B3*, and *ATP12A*.

Analysis of transcripts differentially expressed in HD71Q iPSC lines showed the presence of processes connected with DNA damage response among which *CDKN1A*, *GADD45B*, *DDB2*, and *RAD51* play major roles (Figure 6 and Supplementary Table S8). Other annotations included regulation of establishment of cell polarity, PPAR signaling pathway, negative regulation of transforming growth factor beta receptor signaling pathway, Prader-Willi and Angelman Syndrome, removal of licensing factors from origins, mononuclear cell migration and inflammatory mediator regulation of TRP channels.

The last ClueGO analysis, considered transcripts significantly dysregulated in HD109Q iPSC lines, generated a large cluster connected with p53 signaling pathway (Figure 6 and Supplementary Table S9). Six genes associated with these processes are also in the list of the top 30 dysregulated transcripts in HD109Q iPSC lines. Different processes highlighted during the analysis, which were specific to HD109Q iPSC lines, included Pancreatic cancer, interleukin-3, 5 and GM-CSF signaling, Leishmaniasis, Systemic lupus erythematosus and a large cluster associated to TNF-related factors activation by TP53.

### Genes Dysregulated in 71Q and 109Q HD iPSC Lines Are Also Shared With ESCs, iPSCs, NSCs, and Neurons Obtained in Other Studies

**FIGURE 7:**
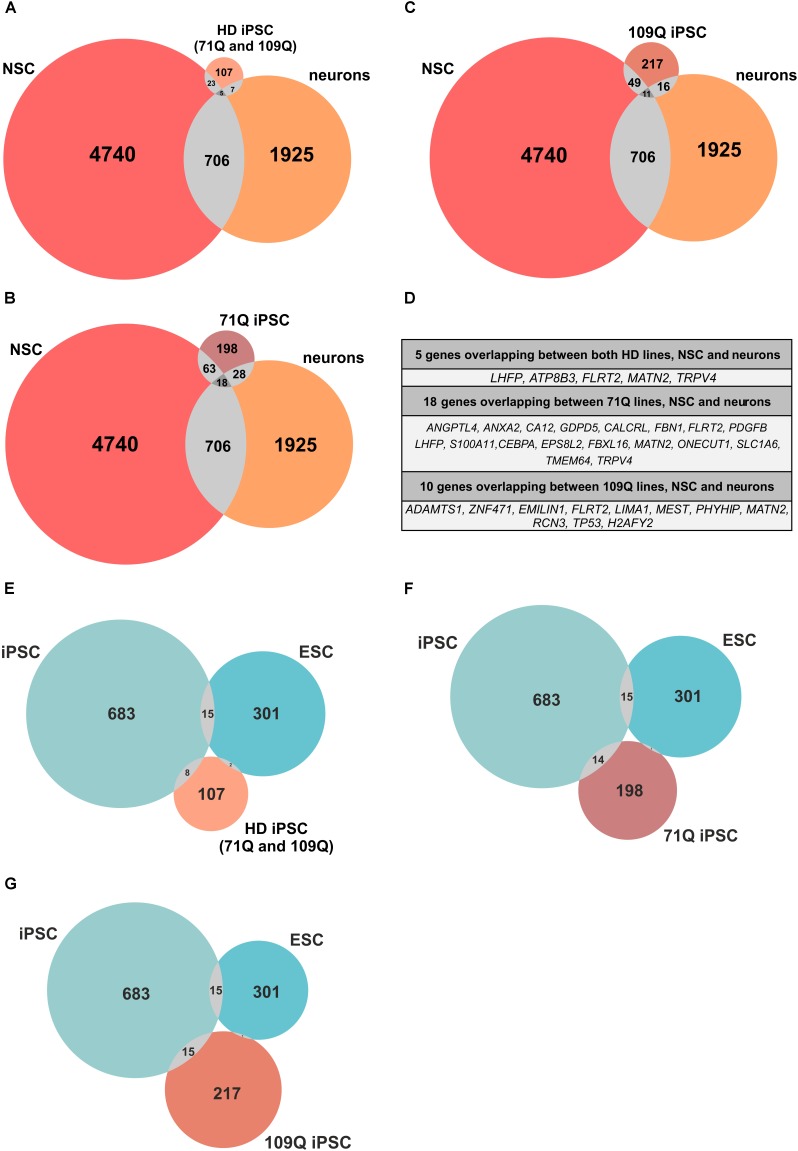
Meta-analysis reveals common genes/proteins for HD lines, ESCs, iPSCs, NSCs and neurons. **(A–C)** Venn diagrams indicate number of genes/proteins included in the meta-analysis that overlap between only two or all three of the analyzed lists of genes. Venn diagrams show number of genes/proteins overlapping between HD iPSC lines and NSCs and neurons. **(D)** Lists of genes/proteins that overlap between all three lists of genes for HD iPSC lines, NSCs and neurons. **(E,F)** Venn diagrams indicate number of genes/proteins included in the meta-analysis that overlap between only two or all three of the analyzed lists of genes. Venn diagrams show number of genes overlapping between HD iPSC lines and ESCs and iPSCs. **(A–C)** Venn diagrams were generated with eulerAPE v3. **(E–G)** Venn diagrams were generated with Meta-Chart.

The performed meta-analysis showed that among genes significantly dysregulated in HD iPSC lines several are shared with iPS-derived or ES-derived NSCs and/or neurons (Figures 7A–D and Supplementary Table S10). Five out of 107 genes found to be altered in both HD lines overlap with genes from neurons. Two of them, *LHFP* and *FLRT2* are strongly downregulated in both HD lines, with log2FoldChange = -4. Among 16 genes which overlap between HD109Q lines and neurons 9 are strongly downregulated in our work, with *TP53* on top of the list. Names of genes overlapping between all three comparison groups were listed in Figure 7D.

The meta-analysis was also performed to establish whether genes altered in our study are also shared with iPSCs obtained in other studies (Figures 7E–G). The analysis revealed that 8, 15, and 14 genes dysregulated in both HD, 71Q and 109Q lines are shared with iPSCs, respectively. Among eight genes which overlap between both HD lines and iPSCs from other studies, *ZNF208* and *ZNF257* were the most dysregulated in our analyses with log2FoldChange 9,24 and 7,15, respectively. Lists of overlapping genes created during the analysis included also those associated with HD-altered neurodevelopmental pathways, like TGFβ (*TGFBI*), as well as p53 pathway (*CDKN1A*, *GADD45B*), cell adhesion (*ANK1*) and calcium signaling (*CALCRL*, *ANXA2*). Lists of overlapping genes are included in Supplementary Table S10. Only four genes overlap between all HD lines and ESCs, including *ACTA1, DPYSL4, HSPA5*, and *PCK2*.

## Discussion

A crucial role of HTT in embryogenesis and nervous system development has been well established (Schulte and Littleton, 2011; Saudou and Humbert, 2016). What is more, an increasing number of reports have begun to asses differential roles of HTT and mHTT during embryogenesis and early neural development processes (Nguyen et al., 2013b), proving molecular changes that occur in HD brains long before the clinical onset of disease symptoms (Wiatr et al., 2018). The reports pointed out several developmental impairments which are due to mHTT, such as the integrity of germ layer specification disruption, precocious oligodendrocyte over neurons maturation, striatal cells vulnerability to death, and dysregulation of Notch signaling pathways (Nguyen et al., 2013b; Molero et al., 2016; Yu and Tanese, 2017). Here, we analyzed iPSC lines from HD patients and unaffected subjects with RNA sequencing and bioinformatics tools. In our study, we aimed to identify alterations in genes and subsequently in biological processes that might be associated with a pathological CAG repeat length. We performed high-throughput analyses of three isogenic iPSC lines from a patient with 71 CAG repeats, three isogenic cell lines from a patient with 109 CAG repeats, and three control lines from two healthy individuals. According to a recent report, the HD109 lines may correspond to ultra juvenile HD and HD71 lines may represent the juvenile HD manifestation (Fusilli et al., 2018). We compared all 6 HD lines to 3 control lines but also each set of 3 isogenic lines to control lines separately to evaluate HD gene expression and to identify early transcriptional changes that occur due to the different range of CAG repeats. As a result, we identified 107 (6 HD lines), 198 (3 HD71Q lines), and 217 (3 HD109Q lines) significantly dysregulated mRNAs in each comparison group. Moreover, we asked the question whether the observed alterations at the mRNA level led to the corresponding changes at the protein level, thereby whether mRNA changes reflect on the functionality of HD stem cells. Our proteomic analyses confirmed similar directionality in dysregulation of protein expression of ZFP30 and TP53. We have previously reported such changes of TP53 protein expression which was highly downregulated in HD109 ultra juvenile HD iPSC lines (Szlachcic et al., 2015, 2017). Bioinformatics analyses of the dysregulated mRNAs and proteins revealed alterations of biological pathways and processes in HD iPSC lines which may have an impact on later neuropathology of Huntington’s disease. These processes related to DNA damage response, p53 signaling pathway, regulation of establishment of cell polarity, and negative regulation of TGFβ signaling pathway.

In previous reports investigating human HD iPSC by RNA-seq the main focus in research on human HD iPSC-derived cells has been put in later differentiation steps, e.g., NSC, however, dysregulated genes in 71Q HD iPSC lines were identified (Ring et al., 2015). In line with the previous report we found a similar number of dysregulated genes and among these genes, 14 have shown dysregulation similarly to Ring et al. (2015) (Supplementary Table S11). Moreover we identified genes associated with HD-altered neurodevelopmental pathways which were dysregulated in human NSCs, like TGFβ (*TGFBI*) and REST (*BDNF*), as well as p53 pathway (*CDKN1A*, *GADD45B*), cell adhesion (*TMEM132C*, *ANK1*) and calcium signaling (*CALCRL*, *ANXA2*) (Ring et al., 2015; HD iPSC Consortium, 2017; Xu et al., 2017). However, BDNF transcript in pluripotent 71Q cells is slightly upregulated, whereas it is depleted at later stages in 71Q (Ring et al., 2015) and other human NSC and neurons (HD iPSC Consortium, 2012) and patients (Zuccato and Cattaneo, 2014). This depletion may be associated with abnormal striatal development and later degeneration. The early upregulation identified in our 71Q iPSC could be the result of the preference of HD pluripotent cells toward differentiation to neural lineages, which was also observed in mouse cells (Nguyen et al., 2013a). However, the majority of identified common genes has not been previously examined in HD. For instance, noteworthy may be the *ZFP57* transcription factor which is the controller of CpG methylation during embryonic development (Strogantsev et al., 2015; Riso et al., 2016; Mohammed et al., 2017).

ClueGO overrepresentation and enrichment analyses for this study showed that many of dysregulated transcripts in HD109Q iPSC lines are involved in DNA damage response and apoptosis, such as *CCND1*, *CDKN1A*, *TP53*, *BAX*, *TNFRSF10B*, *TNFRSF10C*, *TNFRSF10D*, *DDB2*, *PLCB1*, *PRKCQ*, *HSH2D*, *ZMAT3*, *PLK2*, and *RPS27L*. Most of the transcripts were downregulated and their proteins were also showed as direct interactors with TP53 in Induced Network Modules analysis. This may indicate that mHTT interacts with TP53 to alter the level of several TP53 interactors (shown in Figure 4C) influencing the apoptosis. Such disruption in the apoptotic pathway can lead to accumulation of an excessive number of progenitor cells and potential disruption of cell differentiation and production of mature neurons (Pfisterer and Khodosevich, 2017). In addition, HTT effects on cell polarization may result in the generation of incorrect progenitors which need to undergo apoptosis (Godin and Humbert, 2011).

Bioinformatics analysis of transcripts dysregulated in HD71Q iPSC lines revealed that several of them act as transcription regulators during the early multicellular stages of development, such as *ZFP57*, *PIWIL2*, *HIST1H3C*, and *HIST1H2BB*. Significant upregulation of most of these transcripts may lead to a global increase in expression level of genes involved in pathways critical for embryogenesis and early neural development. Interestingly, the mutation in the *HTT* gene may cause precocious neurogenesis, which can lead to subsequent neuropathology (Nguyen et al., 2013a). The analysis of induced network modules (CPDB) in dysregulated mRNAs in both HD lines revealed interactions between genes associated with central nervous system development, axon guidance, signal transduction and migration of cortical neurons during brain development (*BBS2*, *POU6F2*, and *PARK2*). In addition, in all 6 HD lines we found genes such as *DBX2*, *FAM72C*, *TRIM69*, *FLRT2* that were recently reported (still absent annotation as GO terms) as controllers of neuronal development or were found to be enriched in neuronal progenitor cells. DBX2 gene is associated with embryonic and adult neurogenesis while high expression level of this gene can suppress adult neurogenesis (Karaz et al., 2016, p. 2; Lupo et al., 2018). *FAM72C* is one of the human-specific genes enriched in cortical neural progenitor cells (Florio et al., 2018). *TRIM69* is the regulator of brain development demonstrated in zebrafish (Han et al., 2016). What is more, cortical pathology present in Huntington’s disease, may have its molecular onset in downregulation of *FLRT2* gene, which is one of the neural development regulators (Seiradake et al., 2014) and is responsible for regulation of cortical neurons migration during brain development (van Roon-Mom et al., 2008). Furthermore, *FLRT2* is one of the genes which was revealed by our meta-analysis of 9 HD research works (An et al., 2012; Chae et al., 2012; Feyeux et al., 2012; HD iPSC Consortium, 2012, 2017; McQuade et al., 2014; Chiu et al., 2015; Ring et al., 2015; Nekrasov et al., 2016). It overlaps between the analyzed HD iPSC lines and previously reported iPS- or ES-derived NSCs and neurons. Besides the above-mentioned functions of this gene, it has been also reported as one of the potential regulators of rosette neural stem cells (Zhao et al., 2014). Other genes which overlap between the analyzed HD iPSC lines and previously reported NSCs and neurons included *LHFP, ATP8B3, MATN2, TRPV4, ANGPTL4, ANXA2, CA12, GDPD5, CALCRL, FBN1, PDGFB, S100A11, CEBPA, EPS8L2, FBXL16, ONECUT1, SLC1A6, TMEM64, ADAMTS1, ZNF471, EMILIN1, LIMA1, MEST, PHYHIP, RCN3, H2AFY2*, and *TP53.* Except for *TP53*, the gene known to be involved in neurodegenerative disorders, *H2AFY2* seems to be another valuable gene for further studies. H2AFY2, gene encoding histone macroH2A, has been recently linked to Friedrich’s ataxia (Soragni et al., 2015) because of its involvement in *FXN* gene silencing.

Moreover, ClueGO overrepresentation and enrichment analysis identified processes connected with the ErbB signaling pathway. *ERBB3* and *CDKN1A*, genes associated with that GO term, are significantly downregulated in HD109Q iPSC lines. Our findings, along with other recent studies strongly suggest that HD-associated impairments in adult life may result from very early and cumulative embryogenic abnormalities. Studying such early transcriptional changes may help to discover key molecular alterations occurring in HD-affected cells and can thus provide new possibilities for therapeutic and preventive strategies for Huntington’s disease.

## Author Contributions

McF and MrF conceived and designed the RNA-seq experiments. McF and PW analyzed the RNA-seq data. KŚ performed all bioinformatics associated with CPDB and ClueGO analyses of data. KŚ and WS designed and performed the qPCR experiments. MS performed the RNA isolation and assessment. LH was responsible for library preparation. ŁM performed the mass spectrometry analysis of proteins and analysis of proteomic data. WS critically revised the article. KŚ and McF wrote the article. McF and MrF was responsible for concept and obtaining funding.

## Conflict of Interest Statement

The authors declare that the research was conducted in the absence of any commercial or financial relationships that could be construed as a potential conflict of interest.

## Footnotes

1 https://catalog.coriell.org/1/ninds

## Supplementary Material

Figure S1 Graph representing number of reads collected per library during RNA-seq analysis. Each column represents each HD iPSC line.

Figure S2 **(A)**Detection of pluripotency markers in HD iPSC lines (numbered clonal lines: 2, 22, 32 – ND42245; 3, 23, 33 – ND41654; 4, 24, 34 – ND41658; 5, 25, 35 – ND42228; 6, 26, 36 – ND42229; 7, 27, 37 – ND42230; 8, 28, 38 – ND42222; 9, 29, 39 – ND42223; 10, 30, 40 – ND42224). Following markers were selected for the validation: *SOX2* (2–10; product size 338 bp), *NANOG* (12–20; product size 294 bp), *OCT4* (22–30; product size 273 bp) and *LIN28A* (32–40; product size 382 bp). **(B)** Validation of RT-PCR products of all HD lines for reference gene, *GAPDH*. 42–50 – HD iPSC lines in the same order as in **(A)**. **(C)** List of primers used for the detection of pluripotency markers in HD iPSC lines. Primers sequence for *GAPDH* is already in Supplementary Table S1.

Figure S3 **(A)** Heat map representing gene expression patterns of DE genes when comparing HD71Q and HD109Q iPSCs to control iPSCs at an adjusted *p*-value of < 0.05 and baseMean cutoff > 50. Red represents elevated expression while blue represent decreased expression, compared with the row mean. Each column represents each isogenic line. Gene names are shown on the right side of the heat map. As genes identifier type, HGNC symbols were chosen. Heat map clearly shows clusters of genes with the same level of dysregulation in both HD lines and also clusters of genes with the opposite type of dysregulation in HD lines from each patient.

Figure S4 Principal component analysis (PCA) of RNA-seq on HD71Q iPSCs (green), HD109Q iPSCs (red) and unaffected iPSCs (blue).

Table S1 A list of primers used for qPCR validation of RNA-seq results. All primer pairs were designed in Primer3Plus web program.

Table S2 Identifiers from the submitted list of dysregulated genes which were not mapped to distinct proteins in CPDB induced network modules analysis.

Table S3 Lists of transcripts obtained from RNA-seq analysis on 71Q and 109Q HD iPSC lines. Lists contain statistical values for three comparison groups, both HD lines vs. control, HD71Q vs. control, and HD109Q vs. control.

Table S4 Data from proteomic analysis.

Table S5 A list of pathway-based sets containing all the genes, from the submitted list, involved in a specific biochemical pathway. Genes DE in both HD iPSC lines or only in 71Q or 109Q lines were submitted to the analysis.

Table S6 A list of complex-based sets containing sets of genes whose protein products are over-represented in the same protein complex. Genes DE in both HD iPSC lines or only in 71Q or 109Q lines were submitted to the analysis.

Table S7 GO/pathway terms specific for genes dysregulated in both HD iPSC lines. Number of genes associated with the term and the percentage of genes per term are included. Colors of each functional set corresponds with colors of functional clusters shown in Figure 4A.

Table S8 GO/pathway terms specific for genes dysregulated only in 71Q HD iPSC lines. Number of genes associated with the term and the percentage of genes per term are included. Colors of each functional set corresponds with colors of functional clusters shown in Figure 4B.

Table S9 GO/pathway terms specific for genes dysregulated only in 109Q HD iPSC lines. Number of genes associated with the term and the percentage of genes per term are included. Colors of each functional set corresponds with colors of functional clusters shown in Figure 4C.

Table S10 Lists of genes used for and generated from the meta-analysis of differences in genes expression. Genes overlapping between lists, and the genes reported in more than one of the 9 studies included in the meta-analysis, were identified using MS Excel formulas and are shown in properly entitled columns.

Table S11 List of genes similarly dysregulated in Ring et al. publication (Ring et al., 2015).
