# Supplementary figures and images for: Identification of Altered Developmental Pathways in Human Juvenile HD iPSC With 71Q and 109Q Using Transcriptome Profiling
Source: Front Cell Neurosci. 2019 Jan 18;12:528. doi: 10.3389/fncel.2018.00528 (PMC6345698; doi:10.3389/fncel.2018.00528)

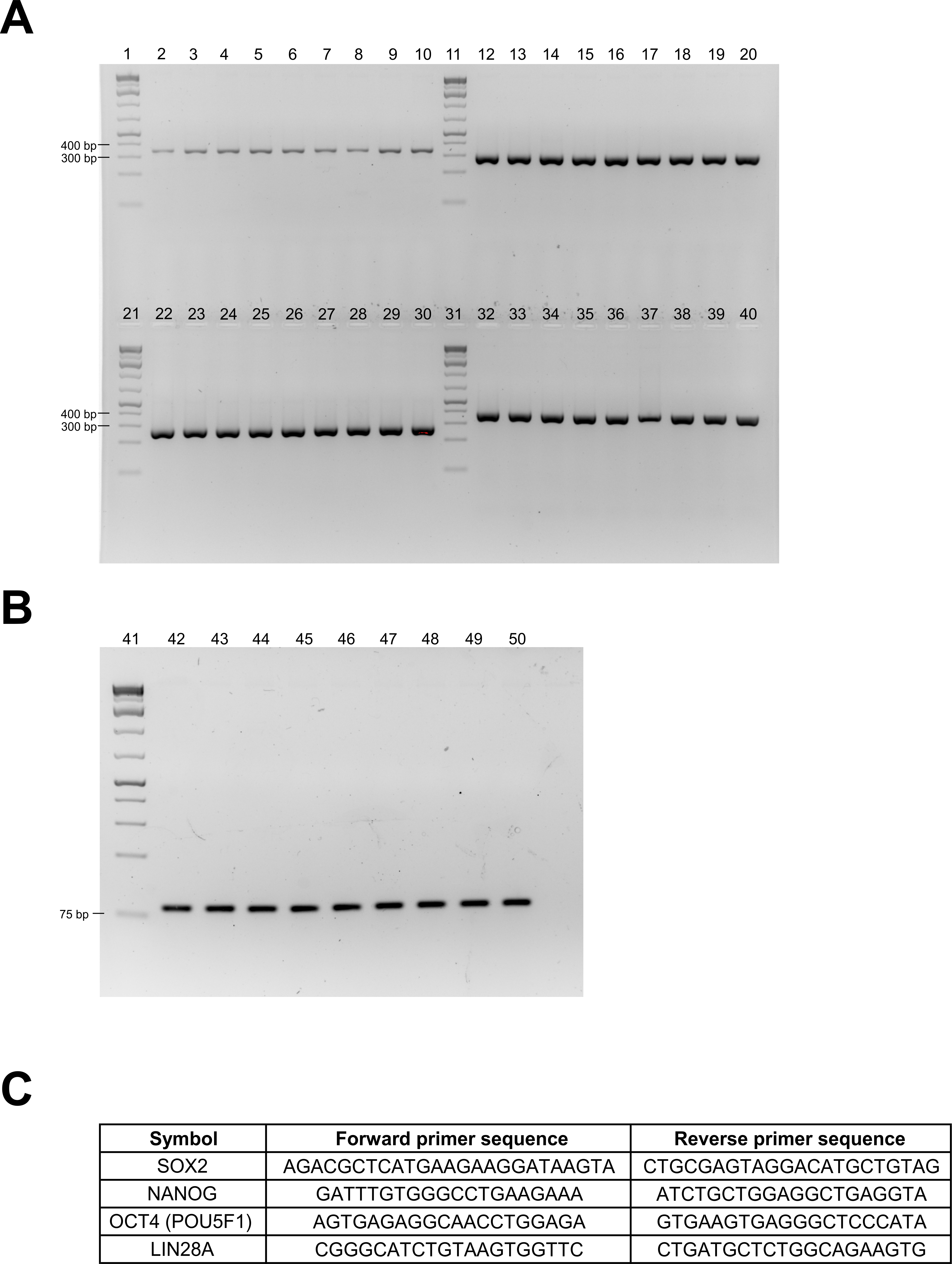

Supplement: Figure S2 — (A)Detection of pluripotency markers in HD iPSC lines (numbered clonal lines: 2, 22, 32 – ND42245; 3, 23, 33 – ND41654; 4, 24, 34 – ND41658; 5, 25, 35 – ND42228; 6, 26, 36 – ND42229; 7, 27, 37 – ND42230; 8, 28, 38 – ND42222; 9, 29, 39 – ND42223; 10, 30, 40 – ND42224). Following markers were selected for the validation: SOX2 (2–10; product size 338 bp), NANOG (12–20; product size 294 bp), OCT4 (22–30; product size 273 bp) and LIN28A (32–40; product size 382 bp). (B) Validation of RT-PCR products of all HD lines for reference gene, GAPDH. 42–50 – HD iPSC lines in the same order as in (A). (C) List of primers used for the detection of pluripotency markers in HD iPSC lines. Primers sequence for GAPDH is already in Supplementary Table S1. [file Image_2.JPEG]

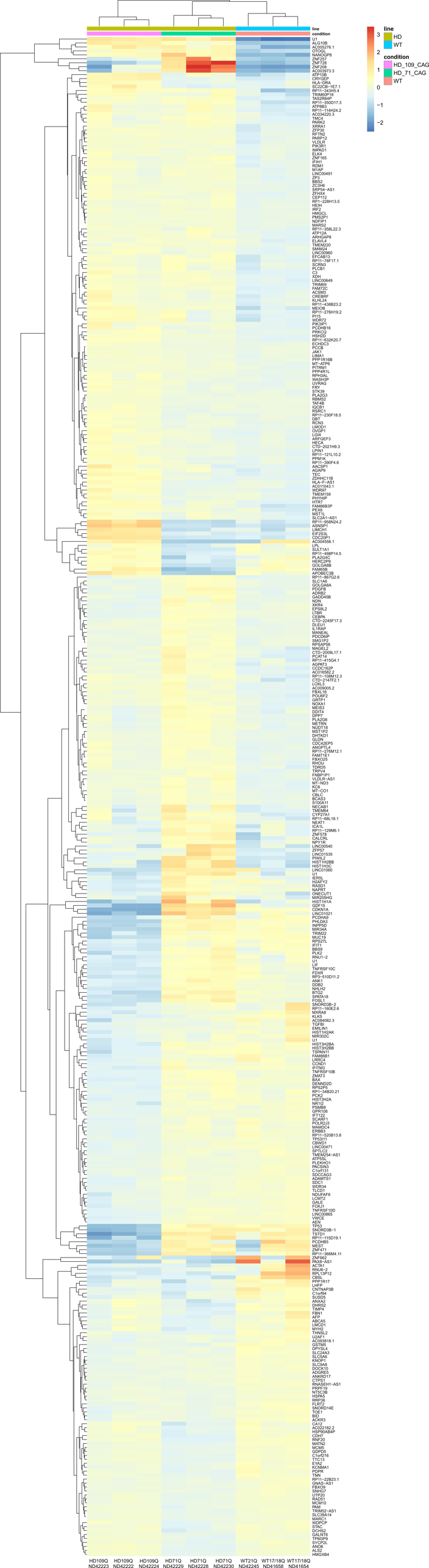

Supplement: Figure S3 — (A) Heat map representing gene expression patterns of DE genes when comparing HD71Q and HD109Q iPSCs to control iPSCs at an adjusted p-value of < 0.05 and baseMean cutoff > 50. Red represents elevated expression while blue represent decreased expression, compared with the row mean. Each column represents each isogenic line. Gene names are shown on the right side of the heat map. As genes identifier type, HGNC symbols were chosen. Heat map clearly shows clusters of genes with the same level of dysregulation in both HD lines and also clusters of genes with the opposite type of dysregulation in HD lines from each patient. [file Image_3.JPEG]

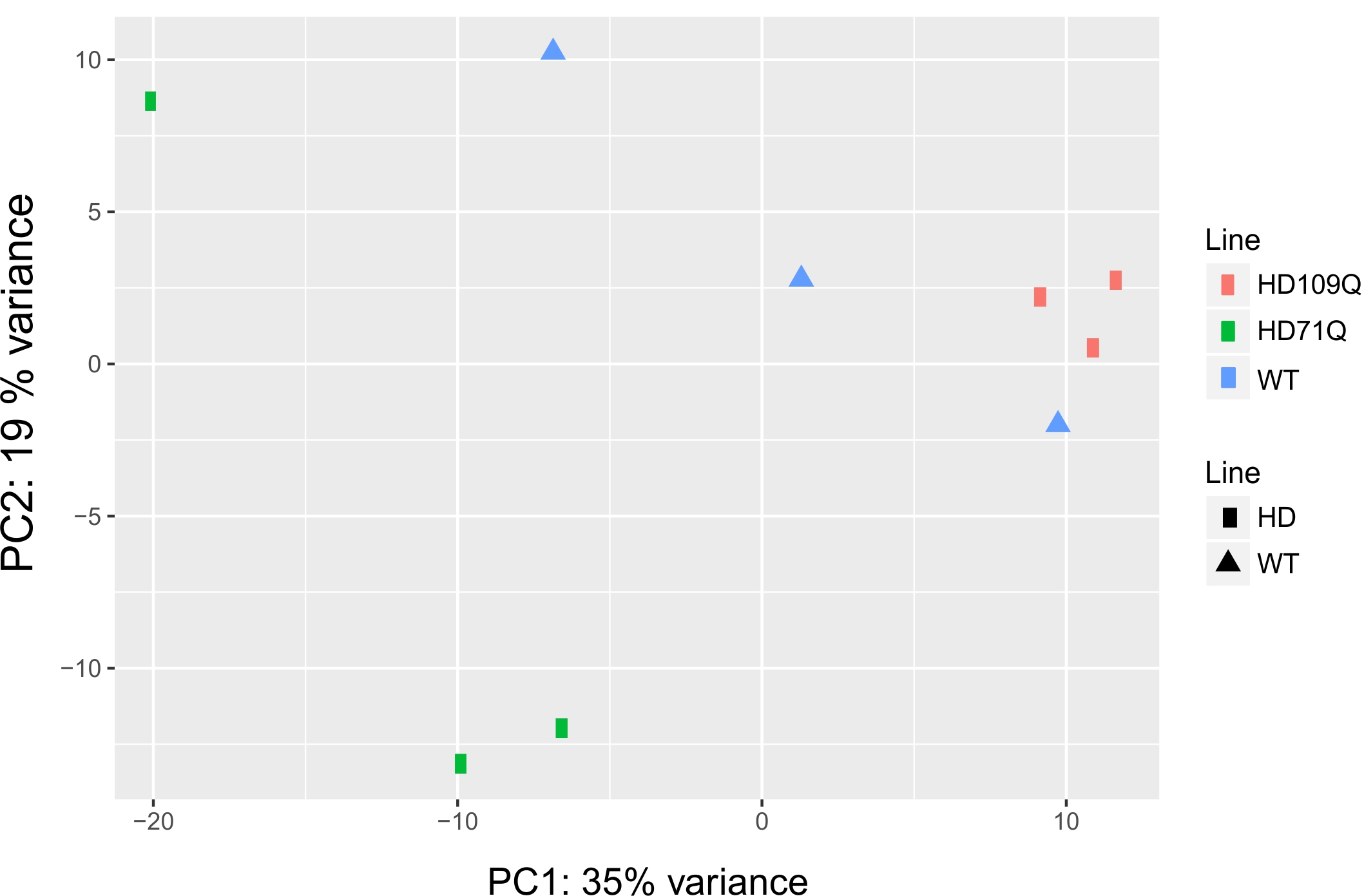

Supplement: Figure S4 — Principal component analysis (PCA) of RNA-seq on HD71Q iPSCs (green), HD109Q iPSCs (red) and unaffected iPSCs (blue). [file Image_4.JPEG]
